# Supplementary material for: Chromothripsis during telomere crisis is independent of NHEJ, and consistent with a replicative origin
Source: Genome Res. 2019 May;29(5):737–49. doi: 10.1101/gr.240705.118 (PMC6499312; doi:10.1101/gr.240705.118)
Supplement: Supplemental Material [file supp_gr.240705.118_Supplemental_file_1.zip › contigs/annotated_contigs/DB82/contig.2.DB82_length_735_mean_cov_10.7591836735.docx]

**DB82_length_735_mean_cov_10.7591836735**

```
TGATTCATGTTAAGAAGGAAAAATATACCTGATCTAAAAAATGTCTTCATTGAATTCAAGCAAATATTTTCTTGTTGATTTGTATTTAG
 >chr11:90495323-90495675 - E=6e-200
TTTACTAAGCCTATAATGACTAATTTAATGGCGCCTAAAATTGCAGTTTATTCTAACAACAGTAAATAGTCAAATAATTGTTTTTTTTT

CCTGGTTAATTAAATTGTAAAGTGCCTTTCTTATATAGCCTTCTTTTGCTTCTCCTTTACTAGTTGCAATCAACAGCATATGCAGATAA

CAATAACTAACCTGTACAATCCAGAGGCTGCTACTATGAACATTATGGACAAGTTTATCACTACTTATATGCATGGGAATA|GGCA|CT
                                                                                  >chr1
GATCAGATTTAGAAATATAACATGTAAACAACCAAACTTTTAGATTAATGCATGTTCTAAATAATACTTTGTGTCTTAATGAGTGTTCA
1:90494342-90494729 - E=5e-218
AATTCTAAATTGATCCTGAGTCTTACACATAAGCAAGATGAAGGGCTAATCAGTCACTACCACCCACCAAGTAGTTCATTCTGAGCTTT

CCTCTTGGGTATACAATTCTGCCTTAATGTGCTATGTTAATTCATGTAATCATGCCTTTGTTCAACCTTCCACCAAGTCTTCATTGCAT

CCTTTCTCTGAACATGCTTCAGTATTCAGTTTGACAACTGAAAGATAAATTTTTGAAAGACATTTATTTTAACAAATTTCTTAACAAAA

CTCTGATTCAGGATACAAATGGAAT
```
